# Unlocking student well-being: the serial mediation of screen time and emotion management ability in the physical activity-life satisfaction link, moderated by health literacy

**DOI:** 10.3389/fpubh.2025.1696358

**Published:** 2025-10-29

**Authors:** Qi Liu, Wei-dong Zhu, Shanshan Han, Hu Lou, Bo Li, Tao Liu

**Affiliations:** ^1^Institute of Sports Science, Nantong University, Nantong, China; ^2^Physical Education and Sports School, Soochow University, Soochow, China

**Keywords:** physical activity, satisfaction with life, emotional management ability, screen time, health literacy, mediation analysis

## Abstract

**Objective:**

The present study aims to explore the influence of physical activity on Satisfaction with Life in a sample of university students. It examines the serial mediating roles of screen time and emotional management ability in the relationship between physical activity and Satisfaction with Life. Furthermore, it explores the moderating effect of health literacy on the path from emotional management ability to Satisfaction with Life.

**Methods:**

The study employed a stratified, cluster, and multi-stage sampling strategy to collect demographic information. Relevant data about university students’ physical activity, Satisfaction with Life, screen time, emotional management ability, and health literacy were obtained through the Wenjuanxing online survey platform. A total of 24,979 valid questionnaires were collected and included in the analysis.

**Results:**

The present research investigated the relationship between physical activity and Satisfaction with Life in university students, focusing on the mediating and moderating mechanisms. The findings indicate that higher levels of physical activity are associated with greater life satisfaction. This relationship is explained in part by two sequential mediators: reduced screen time and improved emotional management ability. Physical activity appears to contribute to lower screen time, which in turn supports better emotional management, ultimately leading to higher life satisfaction. Additionally, health literacy was found to strengthen the connection between emotional management and life satisfaction—students with stronger health literacy skills derived greater satisfaction benefits from their ability to manage emotions.

**Conclusion:**

This research elucidates the influence of physical activity, screen time, and emotional management ability on the Satisfaction with Life of university students. The results suggest that interventions targeting increased physical activity, reduced screen time, and enhanced emotion regulation may effectively promote Satisfaction with Life among this demographic. It is recommended that future interventions integrate evidence-based strategies for fostering healthy screen time practices, emotion regulation training, and comprehensive health education to optimize the physical and mental health of university students.

## Introduction

Satisfaction with Life (SWL) refers to an individual’s stable and generalized cognitive evaluation of their overall life circumstances and core life domains. It not only constitutes a key cognitive dimension of subjective well-being but also serves as a critical indicator for assessing overall quality of life (1–3). From a social adaptation perspective, individuals with higher SWL typically exhibit more pronounced social connectedness and a stronger propensity for prosocial behavior. This positive psychological state facilitates the establishment of healthy interpersonal relationships, thereby fostering a virtuous cycle of social adaptation (4, 5). Relevant research has revealed that elevated SWL may influence student development through multiple pathways, not only enhancing academic performance and increasing engagement in educational environments but also fostering prosocial behaviors (6, 7). Research indicates a positive correlation between higher levels of SWL among university students and more favorable academic expectations, coupled with reduced levels of academic stress (8, 9). Research indicates a positive correlation between higher SWL and improved physical health, particularly in mitigating the risk of chronic diseases. Furthermore, SWL demonstrates prophylactic and therapeutic effects against depression and anxiety, effectively reducing the incidence of these conditions and contributing to better overall health outcomes (10, 11). Research suggests a close association between physical activity and SWL, with the potential for a positive correlation between the two (12). Informed by an understanding of SWL, this study investigates the potential mediating mechanisms through which physical activity may influence SWL.

Physical activity (PA) is defined as any bodily movement produced by the contraction of skeletal muscles that results in a substantial increase in energy expenditure (13). PA is defined as any bodily movement produced by the contraction of skeletal muscles that results in a significant increase in energy expenditure (14–16). Regular PA demonstrably reduces the risk of numerous chronic diseases, concurrently enhancing emotional stability and significantly improving the capacity to manage stress (17). A recent systematic review and meta-analysis confirms a positive association between PA and life satisfaction among adolescents and young adults (18). But a significant number of students experience severely inadequate PA levels upon entering university. According to research by Marcus et al., only 38% of university students engage in regular PA (19). Students who regularly engage in PA exhibit more positive psychological attributes and greater well-being compared to those who participate less frequently (20). Research from the United States indicates that PA, as a positive lifestyle modification, has a significant positive impact on both subjective well-being and SWL in healthy individuals (21). Relevant research indicates a positive correlation between PA and SWL among Chinese university students (22). University students’ screen time is negatively correlated with moderate-to-vigorous PA, suggesting that actively engaging in PA may reduce screen time (23). Despite these benefits, approximately 80% of adolescents worldwide fail to meet the PA recommendations set forth by the World Health Organization (24). Effective interventions targeting screen time reduction to decrease sedentary behavior in university students remain scarce, highlighting a significant gap in health promotion strategies (25).

Screen time (ST), a prevalent behavioral pattern in the digital age, refers to sustained sedentary behavior characterized by exposure to electronic visual display terminals such as televisions, computers, and smartphones (26). Research indicates that excessive ST, particularly engagement with social media, interferes with PA and social interaction, negatively impacting both the mental health levels and SWL of adolescents (27, 28). Moreover, excessive use of such digital media may induce attentional deficits, subsequently contributing to decreased academic performance (29). University students who accumulate 6 h or more of daily ST demonstrate significantly lower mean Grade Point Averages compared to their peers (30). Research originating from the United States indicates that increased nighttime screen use disrupts sleep quality and duration, with adolescent populations exhibiting a particularly pronounced susceptibility to these effects (31). Research indicates that excessive pre-sleep electronic screen use among university students is associated with lower perceived stress coping ability and adverse impact on sleep quality (32). Within the domain of physiological health, prolonged ST, which consequently leads to sedentary behavior, is a significant risk factor for obesity and metabolic syndrome (33). Increased social media usage is strongly associated with elevated levels of loneliness and a reduction in face-to-face social interaction (34). A negative association may exist between ST and emotional management ability (27). This study takes into account the underlying mechanisms of influence between the two factors.

Emotional management ability (EMA) encompasses an individual’s comprehensive capacity to identify, understand, regulate, and express emotions and represents a central focus of contemporary global psychological research (35, 36). Emotions serve as a central driving force for individual behavior, with positive emotions being strongly correlated with SWL (37). Individuals experiencing higher levels of positive affect tend to exhibit a correspondingly greater sense of well-being (38). A Chinese study demonstrates a correlation between physical activity and emotional regulation capacity, with the latter serving as a predictor for adolescents’ life satisfaction (39). EMA significantly impacts the developmental trajectories of university students and exerts a crucial influence on their social adaptability and mental health outcomes (40). Within the context of university life, maladaptive behaviors stemming from inadequate emotional regulation skills and interpersonal difficulties are increasingly prevalent among college students. As they navigate the transition to adulthood, these individuals face multifaceted stressors that, cumulatively, often result in significant emotional distress, potentially triggering a range of mental health issues and behavioral dysregulation. And students with well-developed emotional regulation skills are better equipped to manage negative emotions, promptly adjust their psychological state effectively, and consequently maintain overall psychological and physical well-being (41). Effective emotional regulation skills are crucial for the psychological well-being of university students, as poor EMA is associated with elevated levels of anxiety and depression (42). Students with well-developed EMA also tend to exhibit more effective stress management skills and sustained focus, thereby achieving superior academic performance (43). However, research investigating the role of EMA in the relationship between PA and SWL remains limited and warrants further exploration.

Health literacy (HL) encompasses the capacity of individuals to obtain and comprehend basic health information and services, and to utilize this knowledge to make appropriate health decisions, thereby promoting and maintaining their own health (44, 45). Individuals with higher levels of HL tend to exhibit healthier behaviors and improved mental health, which, in turn, contributes to greater SWL (46). Among adolescents, HL exhibits a significant positive correlation with SWL and demonstrates a predictive effect on the enhancement of EMA, particularly exerting a positive influence on emotion regulation and social skills (47). Individuals with adequate HL possess the capacity to safeguard their own health (48). Enhancing HL can empower students to balance online activities with offline health behaviors better, thereby improving their overall quality of life (49). Research indicates that individuals with higher HL are more knowledgeable about the potential risks associated with excessive ST and are more likely to take steps to reduce their ST. (50) Adolescents with higher HL are more inclined to actively limit recreational ST and to select health-related digital content preferentially[48]. In 2016, the Central Committee of the Communist Party of China and the State Council issued and implemented the “Healthy China 2030” Plan Outline, designating the improvement of national HL as one of its strategic objectives (51). University students represent a crucial segment of the nation’s and society’s future development and are key to achieving the “Healthy China Strategy.” Therefore, enhancing the HL level of university students is of paramount significance.

In our theoretical model, we treated gender, ethnicity, and academic year as controlled covariates, regarding them as fundamental factors that could potentially confound the relationships among the core variables (52). For example, gender may introduce differences across multiple pathways: males generally report higher levels of physical activity, whereas females may be more sensitive to the emotional effects of screen time, potentially leading to a stronger negative impact of screen time on emotional management ability (53). Ethnicity is associated with cultural background and adaptation stress, which may influence individuals’ preferences for physical activity and the extent to which screen time is used as a coping mechanism (54). Academic year reflects varying stress levels among students at different developmental stages—senior students, burdened by academic and employment pressures, may exhibit reduced physical activity and increased screen time, while their emotional management ability may also evolve with psychological maturity (55).

Self-Determination Theory (SDT), proposed by American psychologists Deci and Ryan, aims to elucidate the underlying psychological needs that drive human behavior. The theory posits that human behavior is motivated by three basic psychological needs: autonomy, competence, and relatedness, and further suggests that the satisfaction of these needs promotes individual independence, well-being, and sustained motivation (56). SWL is closely linked to mental health, and PA not only enhances an individual’s physical health but also significantly improves emotional regulation by satisfying psychological needs. HL, as an individual’s capacity to obtain, understand, and utilize health information, is closely associated with EMA and SWL. Research indicates that individuals with higher HL tend to manage their emotions more effectively, adopt more active lifestyles, and thus better cope with academic and life stressors (57). This study is grounded in Self-Determination Theory, which provides a systematic framework for explaining how PA influences the SWL of university students through the chain-mediating effects of ST and EMA, and reveals the moderating mechanisms of HL in this process. This research aims to comprehensively explore the pathways through which PA impacts the SWL of college students, providing a theoretical foundation and practical guidance for promoting their physical and mental well-being.

**Figure 1:**
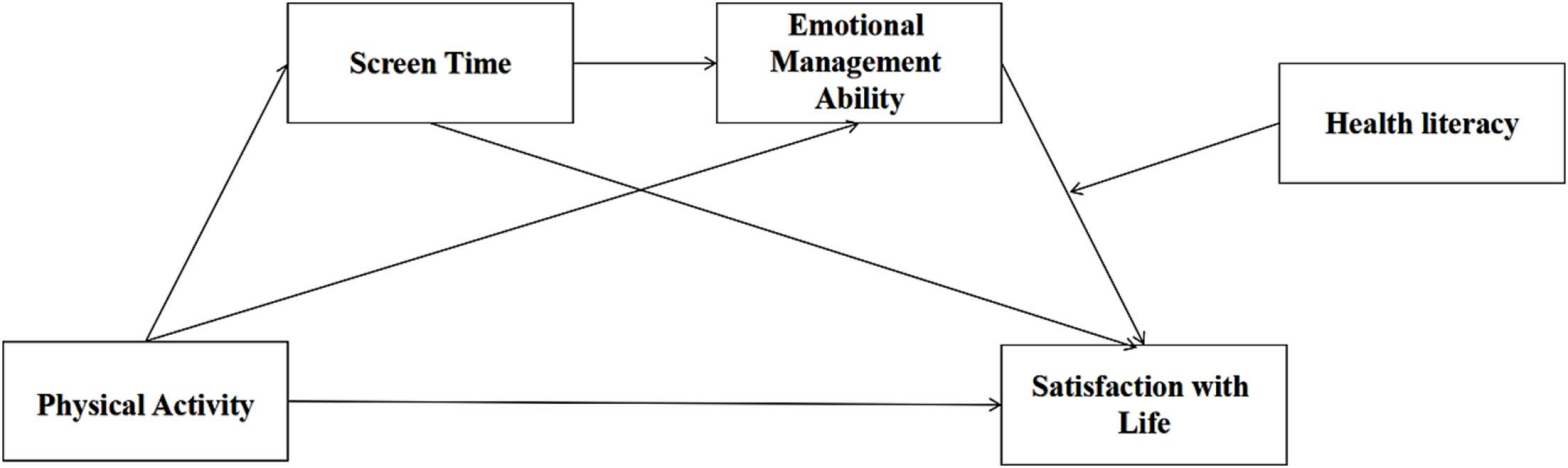
Diagram of the hypothetical model.

Based on the preceding discussion, the following research hypotheses are proposed, with the hypothesized model depicted in Figure 1:

*H1: Physical activity has a significant positive impact on Satisfaction with Life.*

*H2: Screen time exhibits a significant negative mediating effect on the relationship between physical activity and Satisfaction with Life.*

*H3: Emotional management ability exhibits a significant positive mediating effect on the relationship between physical activity and Satisfaction with Life.*

*H4: Screen time and emotional management ability exhibit a significant mediating effect on the relationship between physical activity and Satisfaction with Life.*

*H5: Health literacy exhibits a moderating effect on the relationship between Satisfaction with Life and screen time.*

## Methods

### Participants

This survey primarily employed epidemiological survey methods, utilizing stratified, cluster, and multi-stage sampling techniques to select participants. The study population consisted of undergraduate students enrolled in 104 general higher education institutions in China, specifically including associate’s and bachelor’s degree students, but excluding graduate (Master’s and Doctoral) students. The specific sampling steps are outlined below:

#### Sampling site selection

To ensure the representativeness of the monitored population, each province was allocated an average of three sampling sites. While maintaining an equal sample size across different cities, the specific procedure was as follows: Each province’s/autonomous region’s prefecture-level cities were designated as sampling sites. Among these, the provincial capital city was classified as a “Tier 1” sampling site. The determination of the other two cities was based on the principle of considering the province’s/autonomous region’s geographical location, with one city of average socio-economic development level selected as a “Tier 2” sampling site, and one city with a relatively lower level of socio-economic development chosen as a “Tier 3” sampling site. Sample selection within municipalities directly under the central government did not adhere to the principles above, with random cluster sampling being the primary method, but still adhering to the principle of maintaining three sampling sites.

#### Sampling unit specification

The selection of sampling units primarily considered three aspects: first, the affiliated higher education institutions should be formally registered with the Ministry of Education as complete institutions, including vocational colleges and specialized institutions; second, the units should be able to meet the sampling requirements; and third, the units should have designated personnel responsible for questionnaire distribution and be willing to participate in long-term monitoring. Fourth, the sampled universities should have already completed the return to campus for the autumn semester.

#### Group stratification and sample size

The population was divided into two groups based on sex and then further stratified into four classes based on academic year. Each class was then divided into two categories based on ethnicity. To ensure adequate representation of ethnic minorities in the sample, we paid particular attention to the distribution characteristics of ethnic minority students, selectively choosing institutions with a higher proportion of ethnic minority students and ensuring that at least one college within each institution contained a certain proportion of ethnic minority students. Furthermore, we statistically tested the basic demographic characteristics of the sample using the self-reported ethnicity item in the questionnaire, comparing them with the overall distribution of university students in western China to verify the diversity and representativeness of the sample.

In this study, questionnaire data were collected online, with researchers and supervising professors present during data collection. At the beginning of the study, researchers provided participants with an informed consent form, which clearly outlined the study’s purpose, methods, potential risks, and participants’ rights, ensuring that participants voluntarily participated in the study based on complete knowledge. Participants were assured of the anonymity and confidentiality of their responses both verbally and in the written informed consent form. All participants were informed that the questionnaire would take approximately 12 min to complete and that they could withdraw from the study at any time without any negative consequences. A pilot study was conducted before the questionnaire administration to optimize the questionnaire design based on feedback. Participants’ responses were anonymized, and the acquired sample data were kept confidential to reduce self-report bias. The total sample size available in our database is 40,358. Our data underwent preprocessing. The 24,979 samples presented in this article represent the subset that has been meticulously screened and deemed suitable for the present study. The sample distribution is shown in Table 1.

**Table 1:** Sample characteristics table.

Variant	*n*	%
Gender		
Male	9,796	39.2
Female	15,183	60.8
Ethnicity		
Han	23,093	92.4
Other	1886	7.6
Grade		
Freshman	14,599	58.4
Sophomore	8,108	32.5
Junior	1,640	6.6
Senior	632	2.5
Total	24,979	100.0

### Measurement

#### Physical activity

In this study, PA among university students was measured using the Simplified Chinese version of the Physical Activity Rating Scale (PARS-3), developed by Japanese scholar Yoshio Hashimoto and revised by Liang (58). The Chinese version of the scale is reliable and valid for use with Chinese adolescent populations. The scale examines PA volume across three dimensions: intensity, frequency, and duration of each exercise session, with each dimension containing five options to measure the level of PA participation (59). In completing the questionnaire, higher scores indicate greater PA volume, which, to some extent, reflects the PA participation behavior of university students within a specific time frame. The calculation of raw scores obtained from the questionnaire measurement followed Equation 1.


(1) PAScore=Intensity×(Time−1)×Frequency

The normative classification criteria for Chinese adults using the PARS-3 are as follows: Low PA ≤ 19 points, moderate PA 20–42 points, and high PA ≥ 43 points. The test–retest reliability of this scale is 0.820 (59).

#### Satisfaction with life

In this study, university students’ SWL was measured using the Satisfaction With Life Scale (SWLS) developed by Pavot et al., and its Chinese version translated and revised by Xiong (60, 61). The Cronbach’s α coefficient of the Chinese version of the SWLS was 0.870, indicating that this instrument possesses good measurement characteristics. These values indicate a good fit between the theoretical model and the observed data, suggesting that the SWLS possesses good construct validity.

#### Screen time

In this study, university students’ ST was measured using an open-ended questionnaire. Participants were instructed to recall and report their average daily total time spent on television and computer use over the past month, completing the questionnaire based on their actual situations. This questionnaire has been validated to possess high reliability and validity, demonstrating its feasibility for evaluating ST among university student populations (62). The questionnaire was developed based on the Canadian 24-Hour Movement Guidelines for Adults aged 18–64 years and Adults aged 65 years or older: an integration of PA, sedentary behavior, and sleep. The guidelines recommend that daily ST should not exceed 3 h, and total sedentary behavior should be limited to less than 8 h per day (63). In this study, the questionnaire demonstrated a Cronbach’s alpha coefficient of 0.875, indicating good psychometric properties.

#### Emotional management ability

In this study, university students’ EMA was assessed using the Emotional Intelligence Scale (EIS), originally developed by Mayer and Salovey (35) based on emotional intelligence theory and later translated into Chinese by the scholar Wang. This scale measures individuals’ ability to perceive, understand, express, regulate, manage, and utilize their own and others’ emotions. The EIS evaluates four dimensions: emotion perception, emotion facilitation of thought, emotion understanding, and emotion management. The scale adopts a Likert-type format, consisting of 33 items rated on a 5-point scale (1 = “strongly disagree” to 5 = “strongly agree”), with total scores calculated for analysis (35). The total score ranges from 33 to 165, with higher scores indicating superior individual emotion regulation capabilities (64). The Emotional Intelligence Scale has been extensively utilized and validated by Chinese scholars. Multiple studies have demonstrated its applicability among Chinese university student populations (65, 66). The Chinese version exhibits satisfactory reliability and validity (Cronbach’s α = 0.84) (67).

#### Health literacy

In this study, HL was measured using the Chinese version of the HLS-SF9, which was translated and revised by Sun et al. (68, 69). Assessments were conducted across four dimensions: “Very Difficult,” “Difficult,” “Easy,” and “Very Easy.” The HLS-SF9 used in this study exhibited no ceiling or floor effects. The Cronbach’s α coefficient was 0.913, and the split-half reliability was 0.871, indicating that this instrument possesses good measurement characteristics.

#### Covariate variables

This study selected gender, ethnicity, and academic year as covariates. These three variables have a significant association with the mental and physical health of university students (27, 70). This study selected gender, ethnicity, and academic year as covariates. These three variables have a significant association with the mental and physical health of university students (27). Male university students tend to engage in more PA than female university students, which may contribute to better physical health among males compared to females. Concerning ethnicity, ethnic minority university students, upon entering a new cultural environment, face the impact and stress of cultural differences, which has a significant effect on their psychological well-being. Furthermore, the psychological health status of ethnic minority university students is closely related to their physical health; psychological stress and anxiety may lead to reduced sleep quality, decreased immunity, and so on, consequently affecting overall physical health (71). Regarding grade, first-year students, upon entering university, often experience rapid psychological changes and greater emotional fluctuations, which may lead to poor mental health and, subsequently, affect their overall health.

### Statistical analysis

In this study, data processing was primarily conducted using SPSS 27.0 and Excel software. The data utilized in this research were cross-sectional, collected at a single time point, with predictor, mediator, and outcome variables all assessed concurrently in November 2024 to ensure temporal consistency. The overall process can be delineated into the following steps: (1)Initial data preprocessing was performed using Microsoft Excel software on the data obtained from Wenjuanxing, including retesting or deletion of missing or problematic data. (2) To examine potential common method variance, Harman’s single-factor test was conducted. The principal component analysis yielded six factors with eigenvalues exceeding 1.0, with the most substantial factor explaining 29.197% of the total variance—substantially below the recommended cutoff value of 40% (72). These results provide empirical evidence that common method bias does not pose a substantial threat to the validity of our findings. (3) Statistical differences in PA across gender, ethnicity, and academic year were analyzed using chi-square tests. The strength of associations between categorical variables was determined by Cramer’s V coefficient, which ranges from 0 to 1, with higher values indicating stronger associations. Based on conventional interpretation guidelines, a Cramer’s V > 0.1 suggests a weak association; > 0.3 indicates a moderate association; and > 0.5 represents a strong association between categorical variables (73). Analysis of variance (ANOVA) was conducted to examine group differences in EMA, ST, and HL across gender, ethnicity, and academic year. Effect sizes were calculated using eta-squared (η^2^), with values ranging from 0 to 1. Following Cohen’s conventional benchmarks: η^2^ = 0.01 represents a small effect size, 0.06 indicates a medium effect, and 0.14 constitutes a large effect (74). (4) Pearson correlation analysis was conducted to examine the bivariate relationships among university students’ PA, SWL, ST, EMA, and HL. (5) Mediation analysis was conducted using regression analysis with the PROCESS macro to examine specific mediating effects. Model 87 was selected, with a 95% confidence interval, and the Bootstrap method was employed to analyze the mediation effects and mediating roles in order to explore the mechanisms of action between variables.

## Results

### Descriptive analyses

As presented in Table 2, university students’ PA averaged 17.980 ± 19.856. Male students scored significantly higher than females (*p* < 0.05), and seniors outperformed other academic years. The effect size for academic year (η^2^ = 0.007) was substantially smaller than those for gender and ethnicity. For SWL, the mean score was 23.026 ± 5.699, with no significant differences across gender, ethnicity, or academic year (*all p* > 0.05). However, the effect size for academic year (η^2^ = 0.002) slightly exceeded those for gender and ethnicity. Students’ ST averaged 2.090 ± 0.540, showing no group differences by gender, ethnicity, or academic year. The ethnicity effect size (η^2^ < 0.001) was markedly smaller than those for gender and academic year. In EMA, the mean score was 29.631 ± 4.534, with seniors demonstrating marginally higher scores than other cohorts. The academic year effect size (η^2^ = 0.003) was larger than those for gender and ethnicity. Lastly, HL averaged 27.924 ± 4.219, with males scoring significantly higher than females. The ethnicity effect size (η^2^ < 0.001) was substantially smaller than those for gender and academic year.

**Table 2:** Descriptive statistics of physical activity, satisfaction with life, screen time, emotional management ability, and health literacy among university students, by demographic characteristics.

Variable			Physical activity	Satisfaction with life	Screen time	Emotional management ability	Health literacy
		M	17.98	23.026	2.09	29.631	27.924
		sd	19.856	5.699	0.54	4.534	4.219
Gender	Male	M	26.9	23.104	2.06	29.578	28.238
		sd	24.254	6.017	0.562	5.022	4.655
	Female	M	12.23	22.976	2.11	29.665	27.721
		sd	13.593	5.484	0.524	4.189	3.899
	η^2^		0.13	<0.001	0.002	<0.001	0.004
	*F*		3733.298	3.013	52.663	2.199	89.517
	*p*		<0.001	0.083	<0.001	0.138	<0.001
Ethnicity	Han	M	17.88	23.06	2.09	29.657	27.933
		sd	19.794	5.714	0.539	4.539	4.24
	Other	M	19.26	22.605	2.14	29.321	27.81
		sd	20.556	5.5	0.556	4.464	3.9506
	η^2^		<0.001	<0.001	<0.001	<0.001	<0.001
	*F*		8.481	11.161	14.175	9.537	1.476
	*p*		0.004	<0.001	<0.001	0.002	0.224
Grade	Freshman	M	17.7	22.843	2.08	29.736	27.88
		sd	18.958	5.563	0.523	4.363	4.0582
	Sophomore	M	17.09	23.143	2.12	29.322	27.852
		sd	19.536	5.772	0.559	4.727	4.34
	Junior	M	22.43	23.754	2.13	29.941	28.593
		sd	25.068	6.22	0.566	4.82	4.492
	Senior	M	24.4	23.867	2.18	30.38	28.117
		sd	25.521	6.166	0.578	4.85	5.286
	η^2^		0.007	0.002	0.002	0.003	0.002
	*F*		56.292	19.716	18.249	23.488	15.549
	*p*		<0.001	<0.001	<0.001	<0.001	<0.001

### Correlation analysis

**Figure 2:**
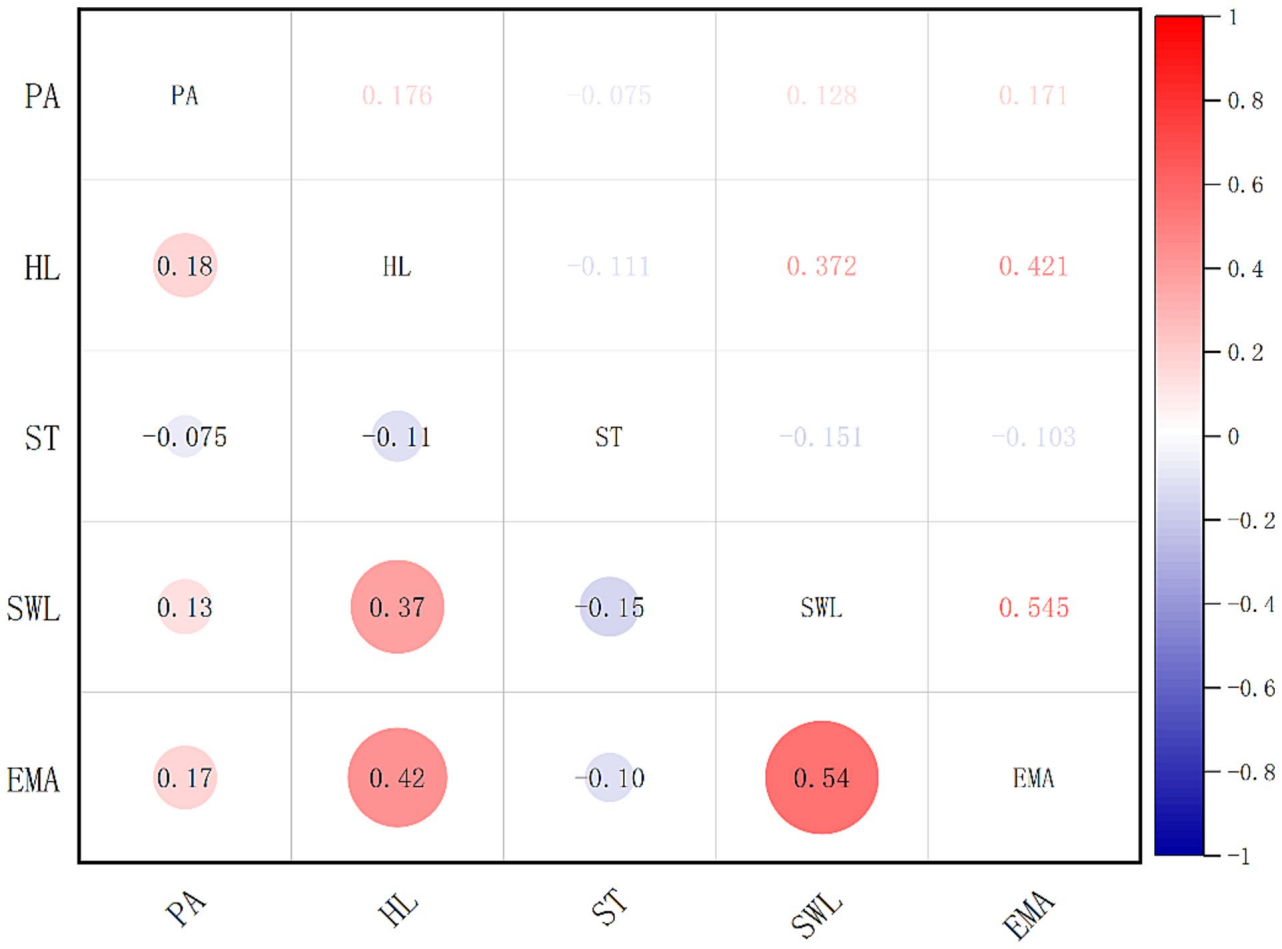
Correlation analyses.

As shown in Figure 2, PA demonstrated significant positive correlations with both HL and its subdimensions, with correlation coefficients ranging from 0.131 to 0.202. Additionally, PA was positively associated with SWL. Significant negative correlations were observed between PA and ST (r = −0.075, *p* < 0.001). PA was significantly positively correlated with EMA (r = 0.171, *p* < 0.001). Significant positive correlations were also found between SWL and HL, as well as its sub-dimensions, with correlation coefficients ranging from 0.308 to 0.372. SWL was significantly negatively correlated with ST (r = −0.151, *p* < 0.001). SWL and EMA were significantly positively correlated (r = 0.545, *p* < 0.001). Significant negative correlations were observed between ST and HL and its sub-dimensions, with correlation coefficients ranging from −0.118 to −0.080. ST and EMA were significantly negatively correlated (r = −0.103, *p* < 0.001). Finally, significant positive correlations were found between EMA and HL, as well as its sub-dimensions, with correlation coefficients ranging from 0.362 to 0.421 (*p* < 0.01).

### Regression analysis

**Figure 3:**
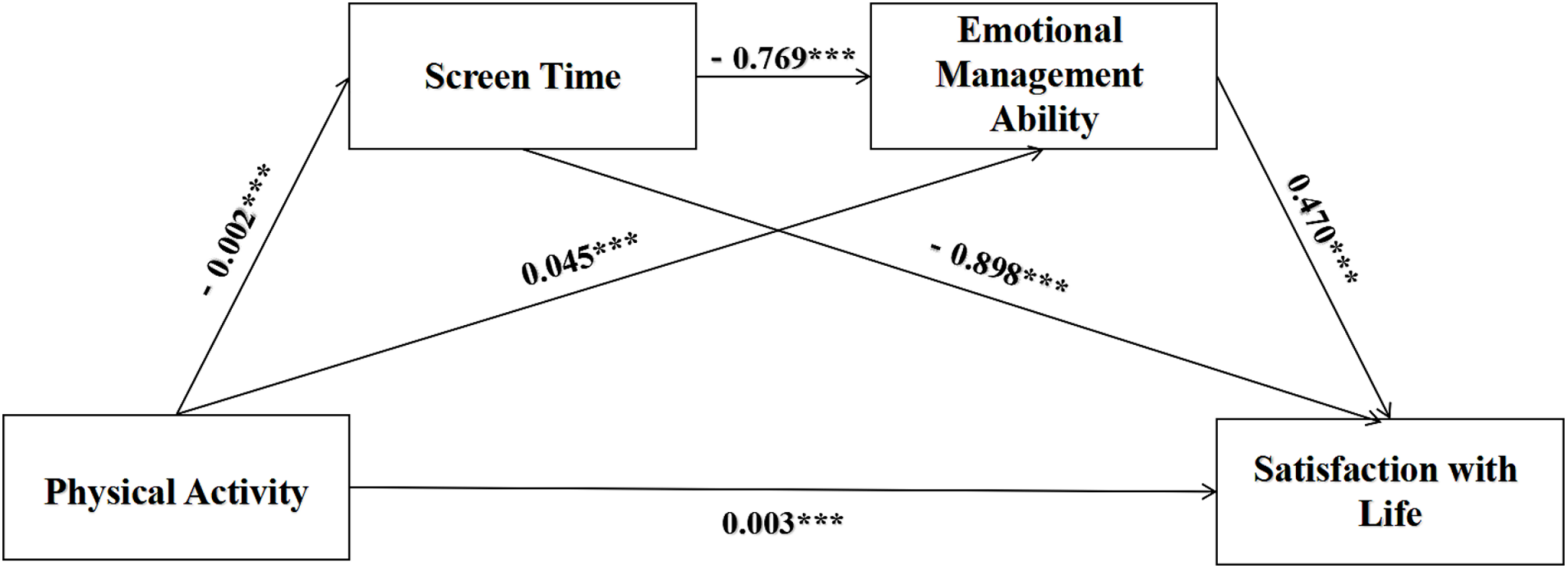
Mediating effect diagram.

In this study, chain mediation analysis was conducted using SPSS 27.0 with the PROCESS macro, Model 6. The Bootstrap method was further employed to test the chain mediation effect, controlling for gender, ethnicity, and academic year. This analysis aimed to examine the chain mediating effect of ST and EMA between PA and SWL. The results, as shown in Table 3, indicated that after controlling for gender, ethnicity, and academic year, PA positively predicted EMA (β = 0.045, *p* < 0.001) and SWL (β = 0.003, *p* < 0.001), and negatively predicted ST (β = −0.002, *p* < 0.001). ST negatively predicted both SWL (β = −0.898, *p* < 0.001) and EMA (β = −0.768, *p* < 0.001). EMA positively predicted SWL (β = 0.470, *p* < 0.001; Figure 3).

**Table 3:** Regression analysis of the relationships among variables in the model.

Regression		Fitting indices			Coeffcient		
Outcome variables	Predictor variables	*R*	*R^2^*	*F*	*β*	*SE*	*t*
Satisfaction with life		0.574	0.331	1544.959***			
	Physical activity				0.003	0.002	2.053***
	Screen time				−0.898	0.055	−16.247***
	Emotional management ability				0.470	0.033	14.056***
	Health literacy				0.103	0.035	2.967***
	Gender				0.042	0.065	0.647***
	Ethnicity				−0.177	0.112	−1.584
	Grade				−0.361	0.041	8.881
Screen time		0.096	0.009	57.817***			
	Physical activity				−0.002	0.0002	−10.507***
	Emotional management				−0.768	0.052	−14.724
	Gender				0.022	0.008	2.978***
	Ethnicity				0.526	0.013	4.081
	Grade				0.037	0.005	7.933
Emotional management ability		0.210	0.044	229.803***			
	Physical				0.045	0.002	29.286***
	Gender				0.780	0.062	12.658***
	Ethnicity				−0.367	0.106	−3.453
	Grade				−0.047	0.037	−1.210

### Test of mediating effects

As presented in Table 4, the 95% confidence interval (CI) for the indirect effect of ST on the relationship between PA and SWL was [0.033, 0.040]. Because this interval did not encompass zero, this indicated a significant mediating effect of ST. Specifically, PA exerted its effect on SWL by reducing ST, which in turn enhanced individuals’ EMA. Furthermore, the 95% CI for the indirect effect of EMA on the relationship between PA and SWL was [0.006, 0.012]. As the interval did not include zero, this demonstrated a significant mediating role for EMA. Moreover, the 95% CI for the chain mediating effect of ST and EMA in the relationship between PA and SWL was [0.0008, 0.0010], indicating a statistically significant chain mediation effect. This suggests that PA, by reducing ST, improved individuals’ emotion regulation, thereby indirectly enhancing their SWL.

**Table 4:** Analysis of the mediating effects.

Effect model	Efficiency value	*BootSE*	*LLCI*	*ULCI*
Total effect	0.037	0.002	0.033	0.040
Direct effect	0.009	0.002	0.006	0.012
Indirect effect	0.028	0.001	0.026	0.030
Physical activity → screen time → satisfaction with life	0.002	0.001	0.002	0.003
Physical activity → emotional management ability → satisfaction with life	0.025	0.002	0.023	0.027
Physical activity → screen time → emotional management ability → satisfaction with life	0.001	0.001	0.0008	0.0010

### Moderation analysis

Using SPSS version 27.0 and the PROCESS macro with Model 87, the moderation effect for each path was analyzed, as shown in Table 5. It presents the results of the moderation analysis examining the moderating impact of HL. In Model 1, with EMA as the dependent variable, and SWL and HL as independent and moderating variables, respectively, the explained variance was 0.3%. The addition of the interaction term in Model 2 increased the explained variance by 2.0%, indicating that the moderation effect, holding SWL and HL constant, contributed 2.0% to the variance explained. In Model 3, the addition of ST and the interaction term increased the explained variance by 30.9%. Finally, in Model 4, with the addition of both ST and the interaction term, the explained variance increased by 33.1%. Furthermore, the significant F-change values across all four models were less than 0.001. The results indicate significant effects of both ST and the moderating variable, HL, on EMA. In the ANOVA results, the *p*-value < 0.001 confirmed the significant moderating effect of HL on the relationship between SWL and EMA. Consequently, HL functions as a significant moderator of the relationship between EMA and SWL. Furthermore, the interaction between HL and SWL significantly moderated the effect of ST on PA (*p* < 0.001). The results indicated that the relationship between EMA and SWL was moderated by HL, supporting Hypothesis H5.

**Table 5:** Table of moderating effect results.

Model	Summary table of the model					ANOVA	
	R^2^	ΔR^2^	df1	df2	Sig. F change	*F*	*p*
1	0.003	0.003	3.000	24,975	<0.001	23.322	<0.001
2	0.020	0.020	1.000	24,974	<0.001	435.469	<0.001
3	0.309	0.309	2.000	24,972	<0.001	5226.357	<0.001
4	0.331	0.331	1.000	24,971	<0.001	808.979	<0.001

## Discussion

Grounded in self-perception theory, this study aimed to elucidate the multifaceted mechanisms underlying the relationship between PA and SWL among university students. By incorporating ST and EMA as mediating variables, the study constructed and validated a chain mediation model linking PA and SWL, with HL serving as a moderating variable. This research provides a basis for higher education institutions to develop scientific educational policies and intervention strategies to promote the holistic development of university students. The study found that the total effect of PA on SWL was 0.036, with a direct effect of 0.009 and an indirect effect of 0.027. This indicates that the impact of PA on SWL involves both direct effects and indirect effects mediated through ST and EMA. While the identified paths and the serial mediation effect were statistically significant, it is important to note that the effect sizes were relatively small. This indicates that while the proposed model is supported by the data, the magnitude of the relationships is modest. This is a common feature in large-scale observational studies. The findings not only deepen theoretical understanding of the relationship between PA and mental health but also provide multifaceted practical implications for psychological health interventions in higher education institutions.

### Descriptive statistical analysis of results

Descriptive analysis revealed no significant differences in university students’ PA levels across gender, ethnicity, and academic year. This finding is consistent with previous research. Significant differences in SWL were observed between genders, which may be attributed to differing definitions of SWL among male and female university students. However, no significant differences were found in SWL across ethnicity or academic year, consistent with prior studies (75). And no significant differences in EMA were observed across gender and academic year, which contrasts with previous research. Several studies have demonstrated significant differences in EMA between genders in secondary school students (76). However, it is plausible that the increasing age and cognitive maturation associated with university students may contribute to a convergence in EMA between genders (77). It is also possible that women are more inclined to utilize cognitive reappraisal strategies for emotion regulation, in conjunction with their heightened emotional awareness (78). Research conducted in Europe and Latin America, however, has revealed significant gender differences in EMA among university students, with female students exhibiting markedly higher capabilities than their male counterparts. This result contrasts with the findings of the present study (79, 80). This divergence from international research may stem from the influence of traditional Chinese cultural values, which advocate for emotional restraint in all genders, thereby suppressing the overt expression of emotions. Research conducted in Europe, the Americas, and Africa has revealed significant differences in EMA across academic years among university students, with senior students demonstrating markedly higher capabilities than their junior counterparts. This finding is inconsistent with the results of our study (81). Western educational systems emphasize the cultivation of independent learning skills and critical thinking, enabling senior students to accumulate more experience in coping with stress. In Africa, the dispersed educational structure may exacerbate this disparity. In contrast, the collectivist culture in China promotes stronger peer socialization effects, which may help explain the divergence in our findings. A significant difference existed for ethnicity, which is consistent with the results from the previous studies (82). University students’ ST did not differ significantly based on gender or ethnicity, a finding consistent with prior research (83, 84). However, differences were observed across academic years, potentially related to varying levels of stress faced by students in different years. Senior students, burdened by greater pressures about survival and employment, may have less free time available, leading to reduced ST. PA among university students significantly and positively predicted SWL, supporting Hypothesis H1. This aligns with previous research (85). These results underscore the importance of considering sociocultural and educational contexts when interpreting findings related to psychological constructs across diverse populations.

### The negative mediating effect of screen time

This study found a negative mediating effect of ST on the relationship between PA and SWL among university students, with a significant indirect effect, thus supporting Hypothesis H2. ST and its sub-dimensions exhibited significant negative correlations with PA and its sub-dimensions, as well as with SWL. This reveals that ST did not act as a bridge between PA and EMA in university students; instead, it exerted a negative influence. ST, as a critical behavioral variable, has been widely studied in recent years, and its impact on individual cognitive development, mental health, and social adaptation is receiving increasing attention (27). This study investigated the mediating mechanisms of ST in the relationship between PA and SWL. The results indicated that the negative mediating effect of ST attenuated the positive effect of PA on SWL. This aligns with previous research, which has consistently demonstrated a significant negative correlation between ST and SWL, indicating a counterproductive impact on individual development and adaptation (27, 86). Furthermore, PA not only promotes a more positive attitude toward health and more effective seeking and utilization of health information, thereby enhancing HL, but also contributes to improved EMA.

### The positive mediating role of emotional management ability competence

This study demonstrated a positive mediating effect of EMA on the relationship between PA and SWL in university students, supporting Hypothesis H3. EMA and their sub-dimensions exhibited significant positive correlations with PA and its sub-dimensions, as well as with SWL. The results indicate that, in the context of PA, enhanced EMA positively contribute to the SWL of university students. This finding is consistent with previous research, which indicates a significant positive association between EMA and SWL, suggesting that improved emotion regulation can assist individuals in more effectively coping with stress and reducing emotional distress. Students with high levels of PA typically exhibit higher levels of physical literacy, including enhanced motor skills, greater self-confidence, and a more positive attitude towards participation (87). They are more inclined to employ proactive lifestyle strategies to cope with psychological stress, exhibiting greater adaptability and capacity for environmental interaction. This not only contributes to an enhanced quality of life but also further reinforces the virtuous cycle between SWL and EMA. Students with high EMA typically demonstrate greater subjective well-being, encompassing higher SWL and more positive emotional states, enabling them to exhibit greater resilience and emotional control in the face of stress (88). Consequently, when students believe they can achieve their desired outcomes through regular PA, their SWL is correspondingly enhanced. This augmented self-confidence makes them more likely to consistently engage in regular PA, which, in turn, influences their EMA and improves emotional stability.

### Serial mediation effects of screen time and emotion management ability competence

The present study demonstrated a chain mediation effect of ST and EMA on the relationship between PA and SWL, thereby supporting Hypothesis H4. Correlation analyses revealed significant negative associations between ST and its sub-dimensions with PA and its sub-dimensions, as well as with SWL and its sub-dimensions. EMA and their sub-dimensions exhibited significant positive correlations with both PA and its sub-dimensions, as well as SWL and its sub-dimensions, further validating the accuracy of the chain mediation model. These findings suggest that PA influences SWL by first negatively impacting ST, which then enhances EMA, and subsequently, that improved EMA positively promote SWL among university students. Self-determination theory emphasizes that human behavior is influenced by both the degree to which basic psychological needs are met and the process of motivational internalization (56). In this study, regular PA effectively reduced individuals’ ST, which consequently led to an enhancement in their EMA.

Aligned with self-perception theory, this research posits that regular PA, through a dynamic process of self-assessment, reshapes individuals’ perceptions of their own capabilities. This reinforcement of positive self-perceptions significantly enhances university students’ subjective evaluations of SWL. Furthermore, this study elucidates the mediating pathway constituted by ST and EMA: PA, by reducing negative screen exposure, and facilitates the improvement of emotion regulation capacity. This finding resonates with Carver’s self-regulation model, which suggests that when individuals decrease passive screen engagement, attentional resources are freed, enabling more effective self-monitoring of emotions (89). HL demonstrated a significant moderating effect within this pathway, aligning with the preeminent role of “cognitive needs” as articulated within Maslow’s hierarchy of needs. Individuals with higher HL are better positioned to translate PA into a structured self-perception, and this cognitive restructuring subsequently strengthens their metacognitive appraisal of life control (90). The promotion of SWL by PA operates through a dual “cognitive-affective” mechanism: it enhances affective experiences via immediate emotion regulation, while also fostering enduring self-efficacy beliefs through the moderating influence of HL. This underscores self-determination theory, wherein the positive effects of PA on SWL are most pronounced when individuals’ needs for autonomy, competence, and relatedness are fulfilled. This research offers a novel perspective on promoting the holistic well-being of university students: in formulating intervention programs, it is crucial to concurrently address ST management, the development of emotion regulation skills, and the recognition and cultivation of HL, thereby maximizing the benefits of PA.

### Health literacy as a moderator in the relationship between satisfaction with life and screen time

The analysis of the moderation effect demonstrated that HL significantly moderates the impact of ST on university students’ SWL. It is well-established that prolonged ST can negatively affect cognitive function (88). Understanding the developmental stage of university students provides additional contextual information for analyzing how factors such as ST influence their SWL. The significant moderating effect of HL on the relationship between ST and SWL observed in this study may be attributed to the fact that students with higher HL possess a greater ability to critically evaluate health-related information, and a higher level of HL may mitigate the negative effects of ST on SWL. Based on these findings, the following recommendations are proposed: (1) Higher education institutions should incorporate HL education into the general education curriculum by offering relevant courses to assist students in improving their information-screening skills, identifying high-quality health content, and reducing ineffective or harmful screen usage. (2) Social media platforms should optimize their content and recommendation algorithms to reduce addictive design features and increase the promotion of health-related information, thereby helping university students establish healthier screen usage habits.

### Limitations

This study is subject to several limitations: (1) The cross-sectional design may prevent definitive causal inferences. (2) The reliance on self-report questionnaires introduces the potential for recall bias. Future research could incorporate objective measurement tools, such as accelerometers, to enhance the reliability and validity of the findings. (3) Social desirability bias may also have influenced participant responses, as individuals may have overestimated their PA levels or underestimated their negative emotions in an attempt to present a more favorable self-image. (4) The study may not have fully accounted for other variables that could influence university students’ SWL. For example, the potential impact of factors such as family economic status, parental rearing styles, social support networks, and individual personality traits on university students’ SWL was not fully explored, which could affect the interpretability of the results.

## Conclusion

This study elucidated the mechanisms through which PA significantly enhances university students’ SWL via both direct and indirect pathways, and validated the moderating role of HL in this process. The findings offer significant implications for health promotion initiatives in higher education institutions: through comprehensive interventions such as optimizing the allocation of sports activity resources, strengthening education on ST management, providing training in emotion regulation skills, and disseminating health knowledge, it is possible to improve students’ perceived health and quality of life effectively. These discoveries provide a theoretical basis and practical direction for formulating scientific health promotion strategies for university students.

## Data Availability

The original contributions presented in the study are included in the article/supplementary material, further inquiries can be directed to the corresponding authors.
